# Time for a Gut Check: A Qualitative Study of Proposed Interventions to Address Gender Inequality in Gastroenterology

**DOI:** 10.1093/jcag/gwad022

**Published:** 2023-09-04

**Authors:** Sowmya Sharma, Holly Mathias, Emma Jones, Courtney Heisler, Noelle Rohatinsky, Kerri Novak, Yvette Leung, Sharyle Fowler, Melaine Kaczur, Laura Targownik, Jennifer L Jones

**Affiliations:** Division of Gastroenterology and Hepatology, Johns Hopkins University, Baltimore 21231, USA; School of Public Health, University of Alberta, Edmonton T6G 2R3, Canada; Department of Digestive Care and Endoscopy, Department of Medicine, Dalhousie University, Halifax B3H 1V7, Canada; Department of Digestive Care and Endoscopy, Department of Medicine, Dalhousie University, Halifax B3H 1V7, Canada; College of Nursing, University of Saskatchewan, Saskatoon S7N 2Z4, Canada; Division of Gastroenterology and Hepatology, University of Calgary, Calgary T2N 4Z5, Canada; Department of Gastroenterology, Faculty of Medicine, University of British Columbia, Vancouver V5Z 1M9, Canada; Division of Gastroenterology, Department of Medicine, Royal University Hospital, University of Saskatchewan, Saskatoon S7N 0W8, Canada; Canadian Hub for Applied and Social Research, University of Saskatchewan, Saskatoon S7N 5B5, Canada; Department of Gastroenterology, University of Toronto, Toronto M5G 1X5, Canada; Department of Gastroenterology, University of Manitoba, Winnipeg R3E 3P4, Canada; Department of Digestive Care and Endoscopy, Department of Medicine, Dalhousie University, Halifax B3H 1V7, Canada

## Abstract

**Background:**

Gender inequalities persist in medicine, particularly in some speciality fields where fewer women are employed. Although previous research has suggested potential interventions to broadly address gender inequality in medicine, no research has focused on interventions in the field of gastroenterology. The purpose of this research was to engage women in the field of gastroenterology in Canada, to identify interventions with potential to be effective in addressing gender inequality.

**Methods:**

A World Café was hosted in 2019 to discuss gender inequality and interventions in gastroenterology. Twelve women employed in the field of gastroenterology (i.e. physicians, nurses, research staff, and trainees) were purposively recruited and participated in the event. The discussion rounds were audio-recorded, transcribed, and thematic analyses was conducted using Braun and Clarke’s principles.

**Results:**

Three key themes identifying potential interventions to address gender inequality in gastroenterology were generated: (1) Education; (2) Addressing institutional structures and polices; and 3) Role modelling and mentorship. Participants indicated that interventions should target various stakeholders, including both women and men in gastroenterology, young girls, patients, and administrators.

**Conclusion:**

Many of the interventions identified by participants correspond with existing research on interventions in general medicine, suggesting that institutional changes can be made for maximum effectiveness. Some novel interventions were also identified, including publicizing instances of gender parity and supporting interventions across the educational and professional lifecourse. Moving forward, institutions must assess their readiness for change and evaluate existing policies, programs, and practices for areas of improvement.

## Introduction

Gender inequality in medicine has been documented in Canada for decades. Although more women are entering medical school in Canada, there remain few women in medical leadership positions.^1–3^ Previous work has also demonstrated that women in academic medicine are less likely to have first- or senior authorship on publications, are less likely to apply for and receive research funding, and experience income disparity, compared to their male peers.^2,4–9^

There are several interventions potentially available for addressing gender-based disparities in medicine. These include promoting peer-mentorship,^10–12^ opportunities for education and professional development,^4,13–15^ improving the transparency and openness of hiring and promotion practices,^12,16–18^ and standardizing pay.^4,19^ Currently, little is known about the extent to which these and other interventions aimed at addressing gender inequality have been evaluated or implemented.

Despite more women entering medical school, several speciality fields in medicine continue to have fewer practicing physicians who are women.^20^ In Canada, approximately 70% of practicing gastroenterologists are men.^21^ A small body of literature has examined gender inequalities within gastroenterology, observing similar inequalities to other fields of medicine. For example, women in gastroenterology experience differences in academic productivity than men, such as fewer number of publications and funded grants.^20,21^ Men also receive more endoscopy time, whereas women gastroenterologists receive more research time.^21^ Further, women earn significantly less (>$100,000) than men in gastroenterology, receive less peer recognition, report greater conflicts with senior colleagues, and have their competency challenged in the workplace.^21^

This project aimed to engage women in the field of gastroenterology in Canada, to identify potential interventions with potential to be effective in addressing professional inequality. To accomplish this goal, we used a qualitative World Café approach to capture the insights and experiences of women in gastroenterology. This paper builds from a larger research study that explored perceived barriers to professional equality among women in gastroenterology.^22^ This paper continues the conversation on professional equality by presenting new data on interventions to address barriers to professional inequality.

## Methods

This study used an exploratory qualitative descriptive design.^23,24^ Participants were recruited using purposive sampling and email invitations were sent to woman-identifying gastroenterologists, nurses, and research staff from Nova Scotia, New Brunswick, Newfoundland and Labrador, Prince Edward Island, and Ontario. A total of 12 individuals participated. In qualitative research, the ideal sample size to reach data saturation is 4–8 participants.^25^ All participants identified as women and included six gastroenterologists, two gastroenterology nurses, three gastroenterology research staff, and one trainee (gastroenterology resident). We purposefully recruited a mix of physicians and other women in the field to understand a range of professional barriers and potential interventions in gastroenterology.

Data collection occurred in 2019 in Halifax, Nova Scotia, during an in-person, full day *Women in Gastroenterology* event. As part of this event, a World Café was structured over 2 hr, with 90 min for three discussion rounds (30 min per topic) and 30 min for debriefing.^26,27^ A World Café is a participatory qualitative research method that integrates seven design principles to support “a conversational process that helps groups engage in constructive dialogue around critical questions…”.^26^ We divided participants into small groups to support robust discussion and allow time for each participant to engage in the discussion. Each focus group concentrated on a specific concern related to gender and gastroenterology. A single open-ended question was used to begin discussion in each focus group. The data presented in this paper are drawn from the focus groups which responded to the following questions:

1. What are the next steps to promoting and improving gender equality in the workplace?
2. What steps are needed for sustainable and system-wide changes, including promotion of diversity in leadership?

Each station had a moderator to encourage conversation through additional prompts. The moderators had previous qualitative research experience and were trained in moderating focus groups. We recorded the discussion rounds. All participants gave informed consent for the World Café to be audio-recorded and for analysis to be conducted. All data were confidential, deidentified, and participants could withdraw at any time.

We transcribed the audio files and all personal identifying information was removed from the transcripts to protect participant privacy. The transcripts were saved as password protected Word files. The transcripts were imported and analyzed in NVivo 12 Pro for Windows software. Inductive thematic analysis was conducted using Braun and Clarke’s principles.^28^ The data analyst read and reread three transcripts to begin the analysis process. The analyst was not part of the data collection process to reduce potential biases of interpretation. They made note of interesting or prominent quotes throughout the selected transcripts. Secondly, they created initial codes for the selected transcripts that centered around the main question for each focus group. The research team met to review the initial codebook. Any discrepancies were discussed among team members to reach consensus. The remaining transcripts were coded using the agreed upon codebook. Codes were organized to generate key themes and subthemes.

## Results

Our analysis generated three key themes concerning potential solutions to promote and improve gender equality in gastroenterology: (1) Education; (2) Addressing institutional structures and polices; and (3) Role modelling and mentorship (outlined in Table 1). These data correspond to key barriers to gender equality that were previously identified by participants.^22^

**Table 1.** Suggested interventions to address gender inequality in gastroenterology.

Type of intervention	Specific interventions
1: Education	• Educate others • Integrate gender and diversity training in medical school curriculum • Offer public training/workshops on women in medicine • Mandate gender and diversity training and ally training for men in gastroenterology • Educate women • Provide salary negotiation training • Increase science, technology, engineering, and mathematic (STEM) education for young girls
2: Institutional structures and policies	• Acknowledge inequalities • Increase institutional acknowledgement of gender issues (e.g. Human Resources processes) • Increase the number of women within gastroenterology who advocate for institutional change • Highlight instances of gender parity within academic medicine • Acknowledge family role(s) • Provide and normalize protected paid parental leave for men and women • Create fair guidance documents for parental leave • Provide childcare supports in the early years • Measure “success” differently between men and women
3: Role modelling and mentorship	• Promote early mentorship of young girls and early career physicians by women leaders in medicine • Identify male colleagues to champion gender equality and provide mentorship

### Theme 1: Education

Participants identified several opportunities where increased education, both for others and themselves, may improve gender equality.

#### Educating others

Most participants suggested that group training on gender and diversity may help address inequality. Many envisioned this training as being a part of mandatory medical school curriculum to create a foundation of understanding before entering practice:

*If you want to reach out to everybody it almost has to become part of the curriculum for that workplace you know? So... there’s obviously a curriculum in medical school with opportunities to introduce there. (Participant 3).*

Some participants also felt that training or workshops should be offered to the public to address stereotypes or “beliefs” about women in gastroenterology, such as women being less capable or knowledgeable than men in gastroenterology. Some participants emphasized the importance of training and education as a preventive tool rather than solely being used after someone has shown discriminatory behaviour, such as movements around cultural sensitivity training. However, a few participants also believed there should be incentives to encourage people, especially men, to take the training, such as requiring it for promotions or making it a part of mandatory regular training.

Some participants suggested opportunities for individual education and training on gender and diversity. A few participants suggested that ally training for men may support their colleagues who are men to be more vocal advocates and allies of women in the workplace.

You have to find a way for this to appeal to men… How do you advocate for women in your workplace? (Participant 11).

#### Educating women

Participants also suggested educational interventions targeting women in gastroenterology. First, participants believed there should be more training on the salary negotiation process to bridge the pay gap.

We don’t get that indication when you’re a fellow. Like you could go to a job and then negotiate and there are different levels or whatever. They just assume that it’s going to be the same for everybody and that they’re even at the same level—there’s not going to be any difference. That’s what you assume (Participant 5).

Some participants also suggested that more emphasis should be placed on science, technology, engineering, and math (STEM) training for young girls to challenge ideas that women underperform in STEM fields or that STEM represents *“things that women aren’t supposed to be good at”* (Participant 11) and encourage them to pursue careers in gastroenterology.

### Theme 2: Addressing institutional structures and policies

Participants acknowledged that interventions targeting institutional structures and policies are needed to address gender inequality, including (1) how inequalities are acknowledged; and (2) how family roles are acknowledged.

#### Acknowledging inequalities

Participants indicated that more needed to be done within their institutions when acknowledging inequalities. Some participants reinforced that institutional barriers were still not properly acknowledged and that better structures needed to be in place to address gender issues. Some participants viewed institutional inequalities as being the work of administrators, such as Human Resources. For example, one participant spoke about a new woman hire who was harassed by male colleagues at a social event with no response from Human Resources (Participant 7). However, some participants suggested that more women in Gastroenterology should be involved in advocating for institutional change, including themselves:

I think when the next female joins our group, because it will happen, I will be the strongest advocate for that to be readdressed and [call a] meeting and say look, this was my experience because I remember...during my maternity leave for which at 8 wk I was postpartum and working part time... (Participant 4).

Taking a more positive inquiry approach, one participant suggested that instances of gender parity should be highlighted in the medical community. She drew attention to the recent graduating class of a local medical school where, for the first time, more women graduated than men.

#### Acknowledging family role(s)

Policies and procedures associated with women’s role in family care work were also identified as key areas for potential interventions. Participants suggested that parental leave should be provided and normalized for all parents so as not to disadvantage women physicians:

When you have children you’ll realize that we... do not have a lot of support for parental leave of any kind... men and women will come back to work very early... basically it’s you can take parental leave if you have enough money in the bank to be able to tide yourself over (Participant 11)

Fair guidance documents were identified as one way to address gender-related workplace policies, including parental leaves, so that decisions regarding leave did not have to be made on a case-by-case basis. Needed supports for parents were identified beyond the childbirth stage. Many participants suggested that childcare supports in their workplace would support their career, especially in cases where their partners were also working or they did not have access to extended family. Some participants also suggested that institutions should measure or define success differently between men and women colleagues, partially because having children has been identified as putting women at a professional disadvantage:

I think if you really want to aim for gender equality you have to identify that the genders are different. Right?... the steps to … become promoted for a man or a woman in such a way, in such a time frame. Well, that timeframe and those steps perhaps are not the right steps [or] timeframe... for a woman (Participant 3)

### Theme 3: Role modelling and mentorship

Participants identified role modelling and mentorship as being a key intervention. Most women identified the timing of mentorship as being important and urged for early mentorship, particularly from women leaders, for both young girls and early career physicians. Several participants also identified opportunities for men to be involved. A few suggested that men who are champions of gender equality should be identified and collaborated with to further advance gender equality:

It’s very important I think to identify male champions as well to support…who have an understanding of this already and who are in the know... or who are open and are supportive to actually work with [women] on this... (Participant 1)

## Discussion

Participants in our study identified a number of potential interventions that could help address gender inequalities in gastroenterology. These included educational interventions, changes to institutional structure and policy, and wider availability of mentorship. Generally, most of the interventions discussed by our participants echoed those proposed in prior research in other fields of medicine. Our research contributes to existing knowledge by moving beyond general medicine and focusing on a specific specialty—gastroenterology.

In recent years, several educational interventions have been successfully piloted and evaluated in the medical workspace. For example, Carnes et al. describe a randomized control trial for a “gender-bias-habit-changing” intervention at the University of Wisconsin-Madison.^13^ Significant changes were noted among faculty members’ “self-efficacy to engage in gender-equity-promoting behaviours,” and “self-reported action to promote gender equity,” as well as perceived “fit” within the department, perceived value of their research, and confidence in raising issues of conflict.^13^ Freund et al. have also underscored the importance of educating women on salary negotiation skills.^4^ Several policy and practice interventions have also been proposed, although most focus on hiring and funding practices.^16,29^ A systematic review suggested there is high-level evidence that changes to institutional hiring practices may help address gender inequality in medicine.^17^ The importance of parental leave policies and work life balance have also been identified.^30^ Finally, research indicates that mentorship, both from men and women, can contribute to the retention of women, develop their leadership potential, and is critical for success in medicine.^10^

The commonality between interventions previously identified and those identified by participants in our research is important, as it suggests medical schools may be able to invest in wide scale interventions which benefit women across disciplines, rather than focusing only on speciality-specific interventions. This finding coincides with a paper by Jagsi et al.’s in which it was stated that institutions must work collaboratively to support women’s success.^31^ Interventions can require significant investment of resources (e.g., time, money, human), therefore collaboration may help maximize the reach of interventions. Evaluating the effectiveness of proposed interventions across multiple specialities is needed to identify context specific considerations and adaptations that may be required.

**Figure 1.**
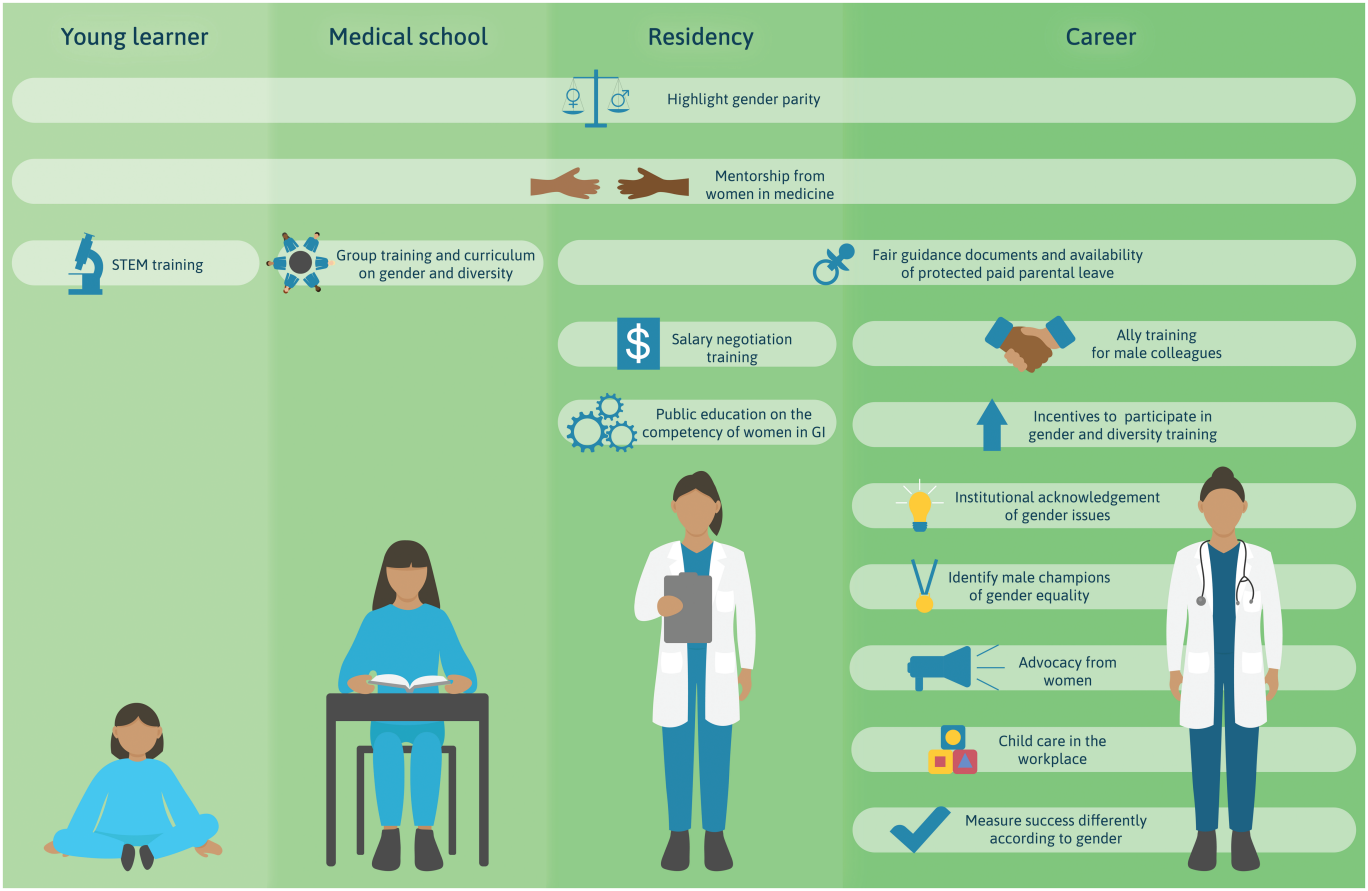
Proposed interventions to address gender inequality in gastroenterology across the lifecourse.

Novel interventions were suggested in our research. For example, having instances of gender parity celebrated and publicized. It was felt that as more information relating to gender parity is disseminated, the greater potential to achieve a societal shift in perceptions of role(s), competency, importance, and acceptance of women in professional and academic leadership positions. This suggestion appears to be new, as previous research has focused on interventions that target negative workplace experiences rather than promoting instances of success. Many participants also proposed a lifecourse approach to interventions, targeting young girls, the public, and women in all stages of professional training and career (see Figure 1). Other intervention research has tended to focus on a few key career milestones (e.g. early career),^6^ whereas our research has identified a range of interventions across the lifecourse.

While analysis of secondarily-collected data has been valuable in delineating the existence of extent of gender-based inequities, qualitative data is critical for better understanding how these inequities are personally experienced, and to better understand the social, environmental, and cultural factors that will impact of the acceptability and ultimate success of a remediative intervention. Using a qualitative World Café approach for this research may have contributed to conversations about lifecourse interventions.

Proposed next steps to “move the needle” on gender inequality in GI and academic and clinical medicine in general include the need for medical departments, faculties, and health systems to make equity diversity and inclusion a priority focus, take an agnostic look at existing policies and programs and compare these to those strategies proposed in this study and the broader medical literature. Employing a Gender-based Analysis Plus lens to existing policies and practices may also help identify challenges to obtaining gender equality.^32^ Additionally, an assessment of institutional readiness for change from a structural, cultural, governance, and organizational perspective is necessary to better understand the potential barriers to successful implementation of institutional interventions meant to address gender inequality in medicine. ^33^ Implementing institutional or policy-based interventions is a particularly complex phenomenon, particularly when a woman is working within an environment in which their professional trajectory is controlled, directly and indirectly, by multiple, large, complex and bureaucratic institutions, often with competing interests, as in the case of medicine. The ability to affect this type of change depends on multiple stakeholders, from multiple institutions, prioritizing the need for these interventions and agreeing to jointly support these interventions. Ideally, leadership in this arena should be national in scope beginning with governments, healthcare systems, medical schools, and local and national physician advocacy organizations.

### Strengths and limitations

To our understanding, this is the first paper to outline potential gender equality interventions specific to the field of gastroenterology, and one of few studies to directly ask women in gastroenterology to identify the interventions they perceive as being important or effective. Asking women directly, who have experienced impact by professional inequity and who have been thoughtful as to the possible contributors to inequity, is a critical form of formal stakeholder engagement that should be used to inform the design of potential interventions. There are also limitations to our research. Our sample size was small and included women working in GI who are not physicians (e.g., nurses, research staff). Although these individuals’ contributions move beyond the experiences of women physicians, we believe it also strengthens the research by providing additional perspectives of gender inequality that are not always captured in clinical medicine research. Further, we did not collect sociodemographic information from participants, which may have informed how other aspects of women’s identity contribute to instances of gender inequality. Eckstrand et al. highlighted the importance of taking an intersectional approach when considering gender inequality in medicine, including the intersections of gender, race, and class.^34^ Future research should explore how intersectionality informs experiences of gender inequality, the design, importance, and appropriateness of potential interventions. Several participants also indicated that incentives may be needed for men to actively participate in gender bias training. Future research may also wish to evaluate the effectiveness of incentives and identify the best incentives to incorporate.

## Conclusions

Our research is the first to ask women in gastroenterology to identify potential interventions to address gender inequality in the workplace. A range of interventions including educational, changes to institutional structures and policies, and mentorship were identified. Future research should focus on better understanding intersectionality as it relates to gender equality and in elucidating institutional and organizational barriers to implementing interventions to reduce gender inequity. Senior medical and health system leadership and colleagues should reflect on barriers to equality for women in gastroenterology, a “gut check” of sorts, and consider the creative design and implementation of some of the proposed interventions. Doing so would signal their support of women’s professional success, which in turn benefits patients, the health system, and society as a whole.

## Data Availability

The data underlying this article cannot be shared publicly due to privacy reasons.
